# Supplementary material for: The Effect of Fertilization on Floristic Composition and Biodiversity of Montane Grasslands (HNV) in the Eastern Carpathians
Source: Plants (Basel). 2025 Dec 26;15(1):80. doi: 10.3390/plants15010080 (PMC12787660; doi:10.3390/plants15010080)
Supplement: Supplementary file 1 [file plants-15-00080-s001.zip › plants-4044958-supplementary.pdf]

Table S1. Average air temperatures (ºC) at Poiana Stampei Meteorological Station (2022-2023).

Specify	Oct.*	Nov.	Dec.	Jan.	Febr.	Mar.	April	May	June	July	Aug.	Sept.	Total 12 months	Vegetation period (IV-IX)
**Multi-year averages**														
Decade I	7.2	4.1	-2.4	-4.2	-2.6	0.4	4.0	9.2	14.3	16.2	17.2	13.8	6.4	12.5
Decade II-a	5.9	1.7	-2.1	-5.2	-3.5	0.4	6.0	9.8	15.1	16.1	16.7	12.7	6.1	12.7
Decade III-a	6.4	-1.1	-2.2	-3.9	-1.2	2.3	7.0	11.9	16.4	17.2	15.8	9.3	6.5	12.9
Monthly average	6.5	1.7	-2.2	-4.4	-2.4	1.0	5.7	10.3	15.3	16.5	16.6	11.9	6.4	12.7
**Agricultural year 2022-2023**														
Decade I	8.2	5.6	0.3	1.5	-7.7	0.6	0.6	8.0	13.4	16.7	16.1	13.2	6.4	11.3
Decade II-a	6.4	2.2	-2.7	1.3	-2.7	0.7	5.2	10.9	13.5	18.2	17.1	14.3	7.0	13.2
Decade III-a	7.4	-0.9	0.4	-2.4	0.3	2.3	5	12.6	15.8	16.2	18.1	13.3	7.3	13.5
Monthly average	7.3	2.3	-0.7	0.1	-3.4	1.2	3.6	10.5	14.2	17.0	17.1	13.6	**6.9**	**12.7**
Multi-year average	6.5	1.7	-2.2	-4.4	-2.4	1.0	5.7	10.3	15.3	16.5	16.6	11.9	6.4	12.8
Deviation	+0.8	+0.6	+1.5	+4.5	-1.0	+0.2	-2.1	+0.2	-1.1	+0.5	+0.5	+1.7	+0.5	+0.1

*** Oct.=October, Nov.=November, Dec.=December, Jan.=January, Febr.=February, Mar.=March, Aug.=August, Sept.=September**

Table S2. Rainfall (mm) at the Poiana Stampei Meteorological Station (2022-2023).

Specify	Oct.*	Nov.	Dec.	Jan.	Febr.	Mar.	April	May	June	July	Aug.	Sept.	Total 12 months	Vegetation period (IV-IX)
**Multi-year averages**														
Decade I	13.6	11.5	14.8	12.4	19.2	11.6	18.7	24.3	31.2	29.9	19.7	19.7	226.6	143.5
Decade II-a	14.6	15.7	12.4	14.8	11.3	11.4	16.5	32.0	40.1	26.8	20.5	20.8	236.9	156.7
Decade III-a	17.2	18.3	17.4	8.1	11.3	10.0	19.2	31.4	50.6	44.6	24.4	22.6	275.1	192.8
**Monthly amount**	**45.4**	**45.5**	**44.6**	**35.3**	**41.8**	**33.0**	**54.4**	**87.7**	**121.9**	**101.3**	**64.6**	**63.1**	**738.6**	**493.0**
**Agricultural year 2022-2023**														
Decade I	4.4	5.3	8.4	13.0	9.3	15.6	38.9	13.9	42.1	18.4	14.6	10.7	194.6	138.6
Decade II-a	15.3	40.4	28.0	27.6	24.2	4.5	10.1	7.5	89.5	39.3	2.0	10.7	299.1	159.1
Decade III-a	9.4	18.4	13.3	9.0	39.4	18.5	48.3	9.5	27.0	78.4	36.2	3.2	310.6	202.6
Monthly amount	29.1	64.1	49.7	49.6	72.9	38.6	97.3	30.9	158.6	136.1	52.8	24.6	**804.3**	**500.3**
Multi-year average	**45.4**	**45.5**	**44.6**	**35.3**	**41.8**	**33.0**	**54.4**	**87.7**	**121.9**	**101.3**	**64.6**	**63.1**	**738.6**	**493.0**
Deviation	-16.3	+18.6	+5.1	+14.3	+31.1	+5.6	+42.9	-56.8	+36.7	+34.8	-11.8	-38.5	+65.7	+7.3

Table S3. Average air temperatures (ºC) at the Poiana Stampei Meteorological Station (2023-2024).

Specify	Oct*.	Nov.	Dec.	Jan.	Febr.	Mar.	April	May	June	July	Aug.	Sept.	Total 12 months	Vegetation period (IV-IX)
							**Agricultural year 2023-2024**							
Decade I	7.9	4.5	-1.2	-2.1	2.1	3.7	9.0	10.2	12.5	17.4	16.7	15.0	8.0	13.5
Decade II-a	6.5	1.4	-1.2	-4.7	1.7	2.2	8.4	9.4	16.5	21.2	19.6	5.4	7.2	13.4
Decade III-a	9.9	-2.0	0.6	-6.1	4.2	5.1	6.8	12.5	19.2	16.7	17.7	9.9	7.9	13.8
Monthly average	8.1	1.3	-0.6	-4.3	2.7	3.7	8.1	10.7	16.1	18.4	18.0	10.1	**7.7**	**13.6**
Multi-year average	**6.5**	**1.7**	**-2.2**	**-4.4**	**-2.4**	**1.0**	**5.7**	**10.3**	**15.3**	**16.5**	**16.6**	**11.9**	**6.4**	**12.7**
Deviation	+1.6	-0.4	+1.6	+0.1	-0.3	+2.7	+2.4	+0.4	+0.8	+1.9	+1.4	-1.8	+1.3	+0.9

Table S4. Rainfall (mm) at the Poiana Stampei Meteorological Station (2023-2024).

Specify	Oct*.	Nov.	Dec.	Jan.	Febr.	Mar.	April	May	June	July	Aug.	Sept.	Total 12 months	Vegetation period (IV-IX)
							**Agricultural year 2023-2024**							
Decade I	11.0	28.0	23.2	16.7	15.3	4.1	10.2	16.8	21.8	79.6	45.2	4.3	276.2	177.9
Decade II-a	24.1	29.6	24.3	36.4	9.4	13.0	26.8	9.2	46.8	28.8	2.0	13.0	263.4	126.6
Decade III-a	22.7	22.4	17.5	7.0	0.1	11.8	15.8	13.8	96.1	26.0	2.2	28.3	263.7	182.2
Monthly amount	57.8	80	65	60.1	24.8	28.9	52.8	39.8	164.7	134.4	49.4	45.6	**803.3**	**486.7**
Multi-year average	45.4	45.5	44.6	35.3	41.8	33.0	54.4	87.7	121.9	101.3	64.6	63.1	738.6	493.0
Deviation	+12.4	+34.5	+20.4	+24.8	-17.0	-4.1	-1.6	-47.9	42.8	33.1	-15.2	-17.5	64.7	-6.3
